# Research Progress, Challenges, and Future Trends of Modified Atmosphere Packaging (MAP) Technology for Food and Agricultural Products: A Bibliometric Analysis (2016–2025)

**DOI:** 10.3390/foods15162875

**Published:** 2026-08-17

**Authors:** Mingyin Hao, Jiahang Liu, Chunhao Kan, Xianzhe Zheng, Chenghai Liu, Yuhan Zhang, Liuyang Shen

**Affiliations:** 1 College of Agricultural Equipment and Energy Engineering, Northeast Agricultural University, Harbin 150030, China; mingy_sept10110@163.com (M.H.); 18249795278@163.com (J.L.); kch1146740211@163.com (C.K.); zhengxz@neau.edu.cn (X.Z.); liuchenghai@neau.edu.cn (C.L.); 2 College of Mechanical and Electronical Engineering, Tarim University, Alar 843300, China

**Keywords:** modified atmosphere packaging, bibliometrics, knowledge mapping, agricultural product preservation, research hotspots

## Abstract

Modified atmosphere packaging (MAP) is a preservation and packaging technology used to extend the shelf life of foods and agricultural products, maintain quality stability, and ensure food safety. To systematically review and summarize the current research landscape, technical challenges, and development trends in the MAP field, this study selected 1568 publications related to MAP from the Web of Science Core Collection (WOSCC) database during 2016–2025 and conducted bibliometric and visualization analysis. The results indicate that research activity in the MAP field has remained robust over the past decade, mainly focusing on four research areas: atmosphere regulation and packaging system design; microbial ecology, safety, and spoilage control; physicochemical deterioration and quality regulation; functional packaging materials and integrated preservation technologies. Countries such as China, Italy, and Spain have demonstrated outstanding performance in terms of publication output and academic influence in the MAP field, forming a solid research foundation in the MAP of perishable foods such as fruit and vegetables, meat products, and aquatic products. Research in the MAP field has gradually shifted from the verification of application effects toward system design and mechanistic analysis. Active packaging, intelligent packaging, bio-based materials, natural functional ingredients, volatile organic compounds, microbial community succession, and quality deterioration mechanisms have gradually become research hotspots, indicating a trend toward precision, sustainability, and functionality. These findings may provide references for future research topic selection, innovative packaging system design, and the development of novel food preservation technologies in the MAP field for foods and agricultural products.

## 1. Introduction

Foods and agricultural products are susceptible to microbial contamination, enzymatic activity, oxidation, respiration, and moisture migration throughout harvesting, processing, storage, transportation, and marketing, resulting in spoilage, nutrient loss, and reduced commercial value [1,2,3]. Consequently, effective storage and preservation technologies are essential for extending shelf life, maintaining product quality, ensuring food safety, and reducing postharvest losses [4,5]. According to the Food and Agriculture Organization of the United Nations, approximately 13.3% of global food is lost between the postharvest and pre-retail stages, with fruits and vegetables exhibiting particularly high loss rates. In addition, approximately 1 billion tons of food are wasted annually at the retail, food-service, and household levels [6]. These figures highlight the continuing need for efficient, safe, and sustainable preservation strategies. Common approaches include refrigeration and freezing [7,8], drying [9], thermal processing [10], chemical preservation [11], vacuum packaging [12], irradiation [7], high-pressure processing [13], and edible coatings [14]. Although these technologies can delay quality deterioration through microbial inhibition, enzyme inactivation, reduced water activity, or the suppression of biochemical reactions [7,15], their application may be constrained by high energy consumption, undesirable changes in sensory or nutritional quality, and concerns regarding consumer acceptance and regulatory compliance [8,9,10,16,17,18,19,20].

In contrast, modified atmosphere packaging (MAP) is a packaging strategy that can extend the shelf life of food products, enhancing safety, and maintaining quality, and is considered one of the important approaches for food preservation [21,22]. The scientific basis of atmosphere-based fruit preservation can be traced to 1927, when Franklin Kidd, Cyril West, and M.N. Kidd systematically examined the effects of regulated gaseous atmospheres on fruit storage [23]. This pioneering work was further developed in a peer-reviewed study published in 1930 [24]. Since then, MAP technology has evolved through several stages, including the theoretical investigation and commercial development of controlled-atmosphere storage, the emergence and expanded application of passive and active MAP, and the integration of functional packaging materials with precise gas-regulation technologies. Over the past decade, rapid advances in novel high-barrier and selectively permeable materials, micro- and nanotechnologies, intelligent sensing technologies, and sustainable packaging materials have ushered MAP into a new era characterized by intelligent, precise, and environmentally sustainable solutions [22,25,26,27,28,29]. The core principle of MAP technology is to regulate the composition of gases such as CO_2_, O_2_, and N_2_ in the packaging system [30], thereby creating a tailored gaseous atmosphere within the package. Such an atmosphere can inhibit microbial growth in products [22], slow oxidative reactions [31], and delay physiological and biochemical deterioration processes in foods [22]. Compared with conventional air packaging, MAP combined with low-temperature storage can reduce respiration, postharvest pathogen development, and spoilage rates in foods under low-O_2_ or high-CO_2_ conditions [32,33,34]. A well-designed MAP system can more effectively maintain the freshness, sensory quality, and commercial value of products during storage and transportation [26,32]. With the growing demand for food preservation and sustainable packaging [22,35], research on the application of MAP in the storage and cold-chain transportation of perishable foods has continued to expand, encompassing fruits, vegetables, meat products, aquatic products, dairy products, and bakery products [22,26,33]. Among these applications, MAP for fruit and vegetables mainly maintains postharvest quality by regulating respiratory metabolism and the gas permeability of packaging films [26,32,33]. MAP for meat products is primarily used for color stabilization, oxidation control, and microbial inhibition [36,37]. MAP for aquatic products focuses mainly on controlling spoilage microorganisms, delaying oxidation, and extending shelf life [31]. In dairy and bakery products, MAP is also used to retard microbial growth, maintain sensory quality, and extend the marketable period of products [35,38,39]. With increasing consumer demand for fresh, minimally processed, and high-quality foods, the application of MAP continues to expand trend [40,41]. In the future, MAP has considerable development potential in reducing the loss of foods and agricultural products, maintaining quality, and constructing sustainable preservation and packaging systems [22,26,33].

The effectiveness of MAP depends on the interactions among gas composition, product characteristics, packaging-material properties, storage temperature, and microbial ecology [32,42]. MAP can be established through active gas flushing, or particularly for fresh fruit and vegetables, through a passive balance between product respiration and film permeability [43]. However, its performance is highly product-specific, and inappropriate gas compositions may cause anaerobic metabolism, off-odors, or inadequate microbial control [34,42,44]. Humidity regulation and condensation control also remain important challenges, especially for high-moisture products [27]. Recent MAP research has increasingly focused on sustainable materials, intelligent packaging, and precise atmosphere regulation, including biodegradable and multifunctional films, equilibrium MAP, anti-fogging and selectively permeable materials, and artificial intelligence-assisted optimization [22,26,27,45,46,47,48,49].

Bibliometric analysis is a quantitative approach for revealing the development, knowledge structure, and emerging trends of a research field [50,51]. Existing bibliometric studies related to MAP have mainly focused on specific commodities, geographical regions, active packaging, or emerging applications [28,52,53,54,55]. However, a comprehensive assessment of MAP research covering food and agricultural products remains limited, particularly regarding collaboration networks, intellectual foundations, thematic evolution, and research frontiers [56]. In recent years, bibliometric studies have been reported in fields such as food packaging and active packaging [52,53,54]. Shi et al. [28] examined the integration of artificial intelligence with MAP for fruit and vegetable preservation, focusing on packaging design, parameter optimization, quality monitoring, and shelf-life prediction. The systematic review and bibliometric analysis of MAP research on horticultural products conducted by Nofiyanti et al. [54] indicated that MAP continues to have research significance in postharvest quality maintenance, shelf-life extension, and packaging material optimization. Isharyadi et al. [55] further pointed out from the perspective of food applications that MAP technology can serve as an alternative packaging method to extend the shelf life of food products and product competitiveness. In addition, the bibliometric study on active food packaging by Dirpan et al. [53] showed that research on active packaging continued to grow from 2000 to 2021 and that research hotspots have emerged around functional materials and food preservation applications. Although these studies provide valuable insights, their analytical scopes are largely restricted to a particular geographical context, commodity category, or emerging technological application. However, relatively few studies have systematically revealed the development of MAP in relation to food and agricultural products, and the visualization analysis of publication performance, collaboration structures, knowledge bases, and thematic changes remains limited [56]. Therefore, using bibliometric methods to systematically analyze the developmental trajectory, research hotspots, and future trends of MAP in this field is helpful for clarifying its knowledge structure.

**Figure 1:**
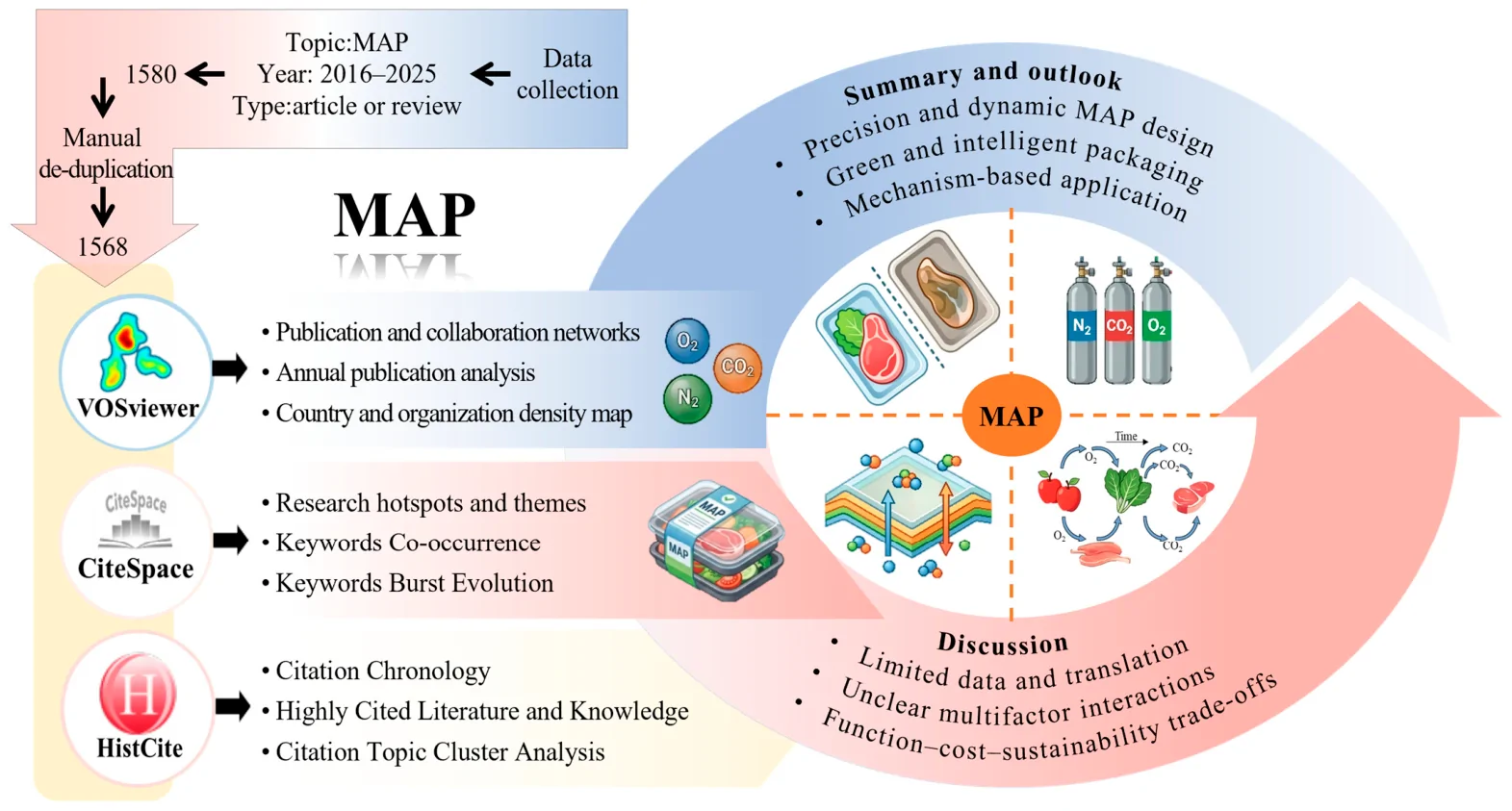
Bibliometric research framework for MAP research.

To address the above research limitations, this study used the Web of Science Core Collection (WOSCC) database as the data source and selected MAP-related English-language publications published worldwide from 2016 to 2025, and bibliometric methods were employed to elucidate the knowledge structure, major research themes, research hotspots, and emerging trends in the field. Bibliometric and visualization analyses were conducted using CiteSpace, VOSviewer, and HistCite, as shown in Figure 1. The specific objectives were as follows: (1) to describe the publication characteristics and collaboration patterns of MAP research from the perspectives of annual publication output, author collaboration networks, country, and institution distribution; (2) to identify research hotspots and development trends through keyword co-occurrence, clustering, and burst analyses; and (3) to further clarify the knowledge structure and research foundation of the MAP field based on highly cited publications and their citation relationships. The main scientific contribution of this study is the identification of an outcome-oriented research core, mechanism-related bridging themes, and rapidly emerging technological fronts, revealing an incomplete transition of MAP research toward integrated and model-assisted packaging-system design.

## 2. Data Sources and Analytical Methods

### 2.1. Data Sources

The WOSCC is a major database commonly used in bibliometric studies. To obtain standardized bibliographic metadata and comprehensive cited-reference information, the literature search and analysis were conducted exclusively using the WOSCC, thereby ensuring the consistency and reproducibility of bibliometric network construction and analysis. It provides broad disciplinary coverage, citation indexing functions, and a reliable basis for literature screening, thereby offering relatively robust data support for analyzing the knowledge structure and evolution of a research field [57]. Many scholars have conducted bibliometric or scientometric studies based on the WOSCC database to reveal the developmental trajectory, hotspot topics, and frontier trends of specific research fields [58]. Therefore, in this study, relevant publications in the field of MAP were retrieved from the WOSCC database. The search query was based on “modified atmosphere packaging” and “MAP”; to ensure both the timeliness of the literature and comprehensive coverage of research advances, the retrieval period was from 1 January 2016 to 31 December 2025; to ensure data consistency and the comparability of bibliometric indicators while maintaining a focus on peer-reviewed scholarly publications in the field of MAP, the document types were limited to articles and reviews; and the language was restricted to English. For each record, information including title, authors, keywords, abstract, publication year, institutional affiliation, citation count, and other relevant bibliographic data was retained. The detailed search strategy, inclusion criteria, and exclusion criteria are presented in Table 1. A total of 1580 relevant publications were initially retrieved. After automatic deduplication using CiteSpace and HistCite, 1568 publications were obtained after deduplication and used as the dataset for subsequent visual bibliometric analysis.

### 2.2. Analytical Methods

The retrieved publications were exported as plain text files in the “Full Record and Cited References” format and imported into VOSviewer 1.6.20, CiteSpace 6.4.R1 and HistCite Pro 2.1 for visual analysis and knowledge mapping. The specific procedures were as follows:

(1) Analysis of annual publication trends, author collaboration, country, and institutional collaboration, and author co-citation relationships. VOSviewer was used to conduct statistical analysis and network visualization of the selected publications. Annual publication trend maps, author collaboration networks, country collaboration networks, institution collaboration networks, and co-cited author networks were constructed. During the analysis, institution names were manually deduplicated to improve the accuracy of the mapping results. The counting method was set to full counting, and representative nodes were selected based on thresholds for the minimum number of publications or minimum citation count.

(2) Identification of research hotspots, thematic structures, and evolutionary trends through keyword co-occurrence, burst detection, clustering, and timeline analysis. CiteSpace 6.4.R1 was used to perform keyword co-occurrence analysis, burst detection, cluster analysis, and timeline analysis, to identify research hotspots, frontier topics, and stage-specific evolutionary characteristics in the MAP field. The parameters were set as follows: the time span was from 2016 to 2025, with a one-year time slice, the node type was set to “Keyword”, and node selection was performed using the modified g-index criterion with a scaling factor of k = 25. Network pruning was conducted using pruning methods including Pathfinder, pruning sliced networks, and pruning the merged network, while the remaining parameters were kept at their default settings. To avoid incorrectly treating similarly worded but semantically distinct terms as synonyms, which could lead to conceptual confusion and the loss of information on specific research themes, the keywords were not manually deduplicated or merged.

(3) Analysis of the local citation network of the included publications using HistCite. The Local Citation Score (LCS) was used to evaluate the local citation influence of publications within the study sample, while the Global Citation Score (GCS) was used to measure their overall citation impact in the WOSCC database. On this basis, the Graph Maker function was applied with the settings “Select by = LCS” and “Limit = 20” to identify core publications with high local citation counts. A chronological citation map was then generated to visually present the citation relationships among key publications and the evolutionary trajectory of the knowledge base in the MAP field.

## 3. Publication Characteristics and Collaboration Network Analysis

### 3.1. Annual Publication Analysis

**Figure 2:**
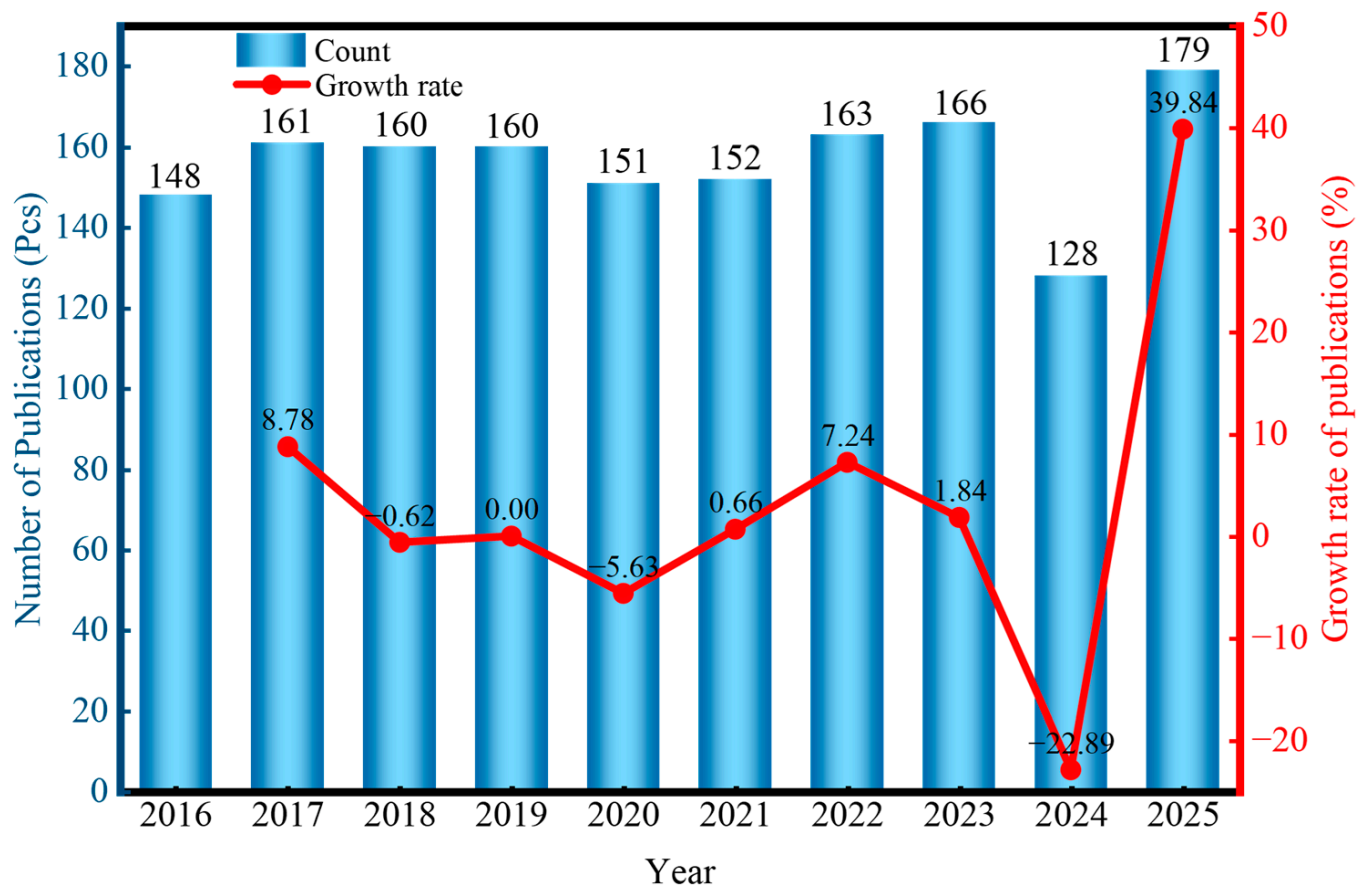
Annual distribution of MAP publications indexed in Web of Science.

The annual publication output in the field of MAP can reflect the development trend of this research area. As shown in Figure 2, a total of 1568 MAP-related publications were published from 2016 to 2025, with an average annual output of 1568 publications. Overall, the publication output showed a fluctuating but generally upward trend. Specifically, from 2016 to 2019, the number of MAP publications remained relatively stable, with 148, 161, 160, and 160 papers published in each year, respectively. In 2020 and 2021, the number of publications was 152 and 151, respectively, representing a slight decrease compared with the previous period, but the overall fluctuation was limited. After 2022, the publication output showed an upward trend again. Although the number of publications declined to 128 in 2024, the overall pattern still indicates a gradual increase in research output in the MAP field, and the annual output reached the highest level of the study period in 2025.

From the perspective of annual growth rate, the changes in 2018, 2019, 2021, and 2023 were relatively small, with rates of -0.62%, 0.00%, 0.66%, and 1.84%, respectively, indicating that publication output remained relatively stable in these years. In contrast, the magnitude of change increased in 2017, 2020, and 2022, while the largest changes occurred in 2024 and 2025, reaching 2.89% and 39.84%, respectively. This suggests that the publication output showed relatively strong fluctuations during the study period.

Overall, from 2016 to 2023, the MAP field developed relatively steadily, with the annual number of publications ranging from 148 to 166, indicating a solid knowledge base and sustained academic attention in this field. However, from the perspective of absolute publication output, the research scale of the MAP field remains limited, suggesting that it is still a relatively niche or highly specialized research area. The number of publications in 2020 and 2021 decreased compared with previous years, which may have been due to research disruptions caused by the COVID-19 pandemic [59,60]. The publication output in 2024 and 2025 showed a fluctuating trend, first decreasing and then increasing, reflecting that the field may be in a transitional stage of the emergence of new research themes and the formation of emerging hotspots. On the one hand, MAP research remains closely associated with practical demands such as food preservation, quality maintenance, shelf-life extension, and cold-chain logistics [26]. On the other hand, advances in packaging materials, quality detection, microbial analysis, intelligent monitoring, and data modeling technologies [27,61] have provided support for the expansion of related research and contributed to the marked rebound in publication output in 2025. This indicates that MAP, as an important technological direction in the field of food preservation, still maintains a high level of research activity and has shown sustained development potential in recent years.

### 3.2. Author Collaboration and Co-Cited Author Analysis

**Figure 3:**
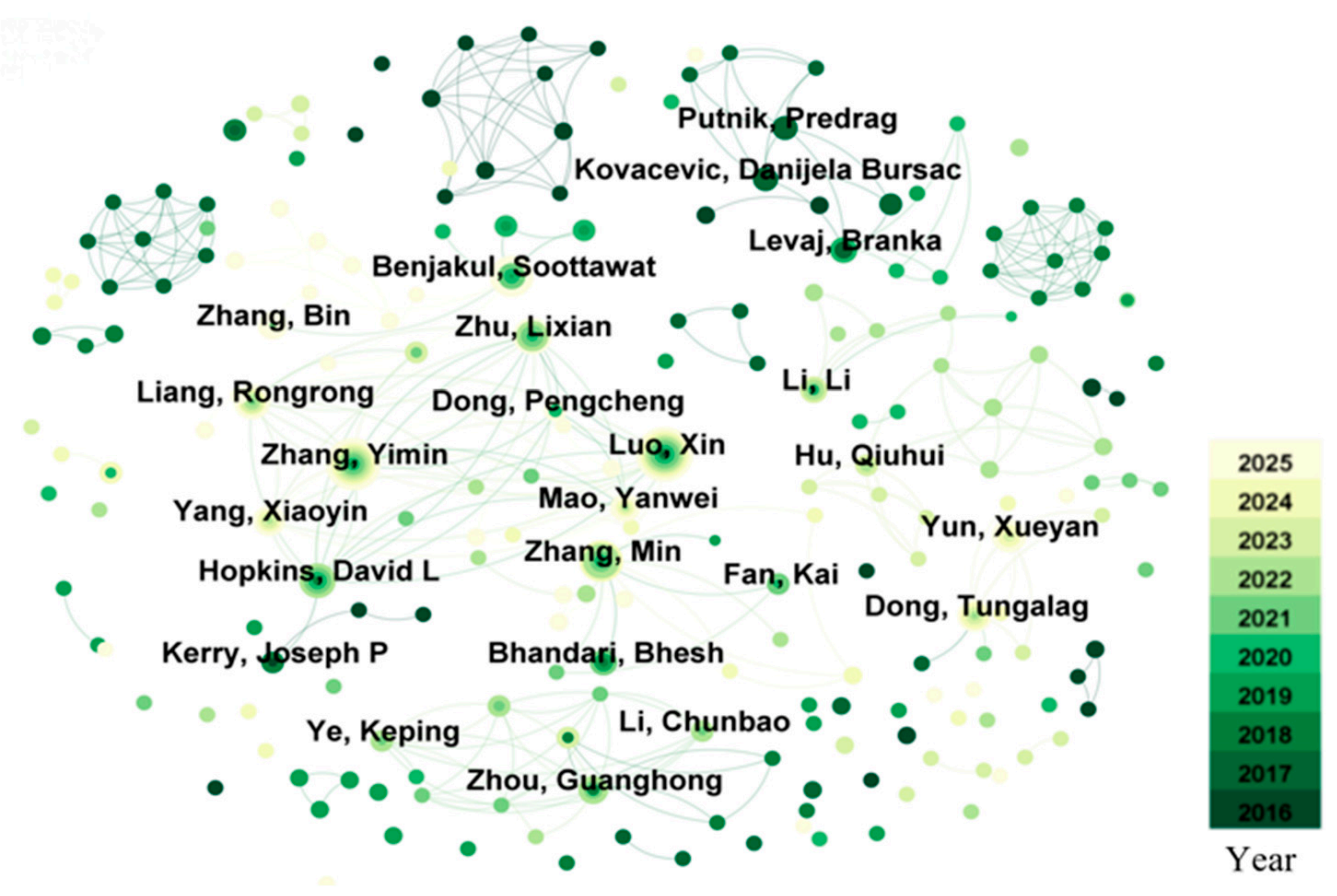
MAP collaborative author relationship map.

In the present study sample, a total of 126 authors published at least five publications in the MAP field, indicating relatively high research productivity and constituting a core author group. Figure 3 shows the author collaboration network constructed based on this core author subset. In the figure, the node size represents the number of publications by each author, while the links reflect the collaborative relationships among authors and their strength. Figure 3 reveals the collaboration patterns and network structure among major researchers, showing that 47 authors formed clear collaborative connections. The structural characteristics of the collaboration network of the MAP field are presented in Table 2. Zhang Min had the highest publication output in the MAP field, with 29 publications. Focusing on MAP, Zhang Min combined coating, ultrasound, light treatment, electric fields, active substances, and other emerging technologies for the preservation of fruit and vegetables, meat, and aquatic products. The preservation effects and storage quality of MAP and its synergistic technologies were evaluated through multidimensional assessment systems, including microbial indicators, physicochemical properties, flavor compounds, and freshness indicators [62]. Zhang Yimin ranked second with 27 publications and Luo Xin with 25 publications. Zhang Yimin has long focused on the optimization of gas composition for beef preservation and found that reducing the O_2_ concentration in MAP systems from 80% to 50% while increasing CO_2_ to 40% could effectively inhibit the growth of spoilage bacteria while maintaining beef color stability [63]. Subsequently, this research further expanded from gas-ratio optimization to the effects of different packaging systems on oxidative stability and product suitability. Luo Xin compared the effects of 80% O_2_-MAP, 50% O_2_-MAP, CO-MAP, and vacuum packaging on the storage quality of highly marbled beef under simulated retail storage conditions at 4 °C [64]. The study revealed that although a high-oxygen environment favored the formation of an initially bright red color, it accelerated lipid and protein oxidation, leading to browning and quality deterioration during later storage. In contrast, CO-MAP and vacuum packaging effectively inhibited oxidative reactions and microbial proliferation, with CO-MAP showing the best performance in maintaining meat color stability.

Co-cited author analysis can reveal the core contributors and their academic influence within the knowledge base of the MAP field. In this study, 33,093 co-cited authors were identified, among whom 484 authors were cited more than 20 times. As shown in Table 3, Adel A. Kader had the highest citation count, with 220 citations, indicating that his research has had an important foundational influence in the MAP field. Adel A. Kader systematically investigated the physiological responses and tolerance ranges of fresh fruit and vegetables under low-O_2_ and high-CO_2_ conditions, clarified the potential advantages of MAP technology in delaying ripening, inhibiting respiration, and maintaining quality, and highlighted the mechanisms by which inappropriate gas parameters may induce anaerobic metabolism, off-odor formation, and physiological disorders [65]. On this basis, Adel A. Kader explained the design principles of MAP from the perspectives of gas diffusion, epidermal resistance of fruit and vegetables, film permeability, active or passive atmosphere modification, and the formation mechanism of equilibrium atmospheres. This promoted the transition of MAP research from empirical packaging trials to rational design based on the physiological characteristics of fruit and vegetables and the principles of gas transfer, providing a theoretical foundation for technological optimization and application research in the MAP field [65].

Morten Sivertsvik ranked second, with 191 citations. His research mainly focused on the preservation of aquatic products using MAP, particularly examining the effects of different gas compositions combined with technologies such as soluble gas stabilization and superchilling on food shelf life, microbial safety, and sensory quality [31]. This line of research was further extended to the analysis of spoilage microbiota and the modeling of packaging systems [66,67], showing strong systematic and practical value.

The authors ranked third through tenth had citation counts ranging from 117 to 151, representing different research directions. Oluwafemi James Caleb and Shukadev Mangaraj mainly promoted the development of MAP for fruit and vegetables from respiration regulation and gas transfer toward active, intelligent, and model-based design [33,68]. Lone Gram and Paw Dalgaard focused on spoilage microorganisms, specific spoilage organisms, and shelf-life prediction in MAP aquatic products, thereby advancing this field toward microbial ecology and predictive modeling [31,69]. Kenneth W. McMillin and Marianne N. Lund mainly conducted research on color maintenance, oxidative stability, and quality deterioration mechanisms in MAP meat products [36,70]. Danilo Ercolini introduced microbial community analysis methods into MAP food research [71], promoting the shift of MAP studies from the macroscopic evaluation of preservation effects toward microbial mechanism analysis.

Overall, these highly co-cited authors established several major knowledge streams in MAP research, including commodity physiology and packaging design, aquatic-product preservation, meat-quality regulation, and microbial ecology.

### 3.3. Publishing Countries and Institutional Collaboration Distribution

**Figure 4:**
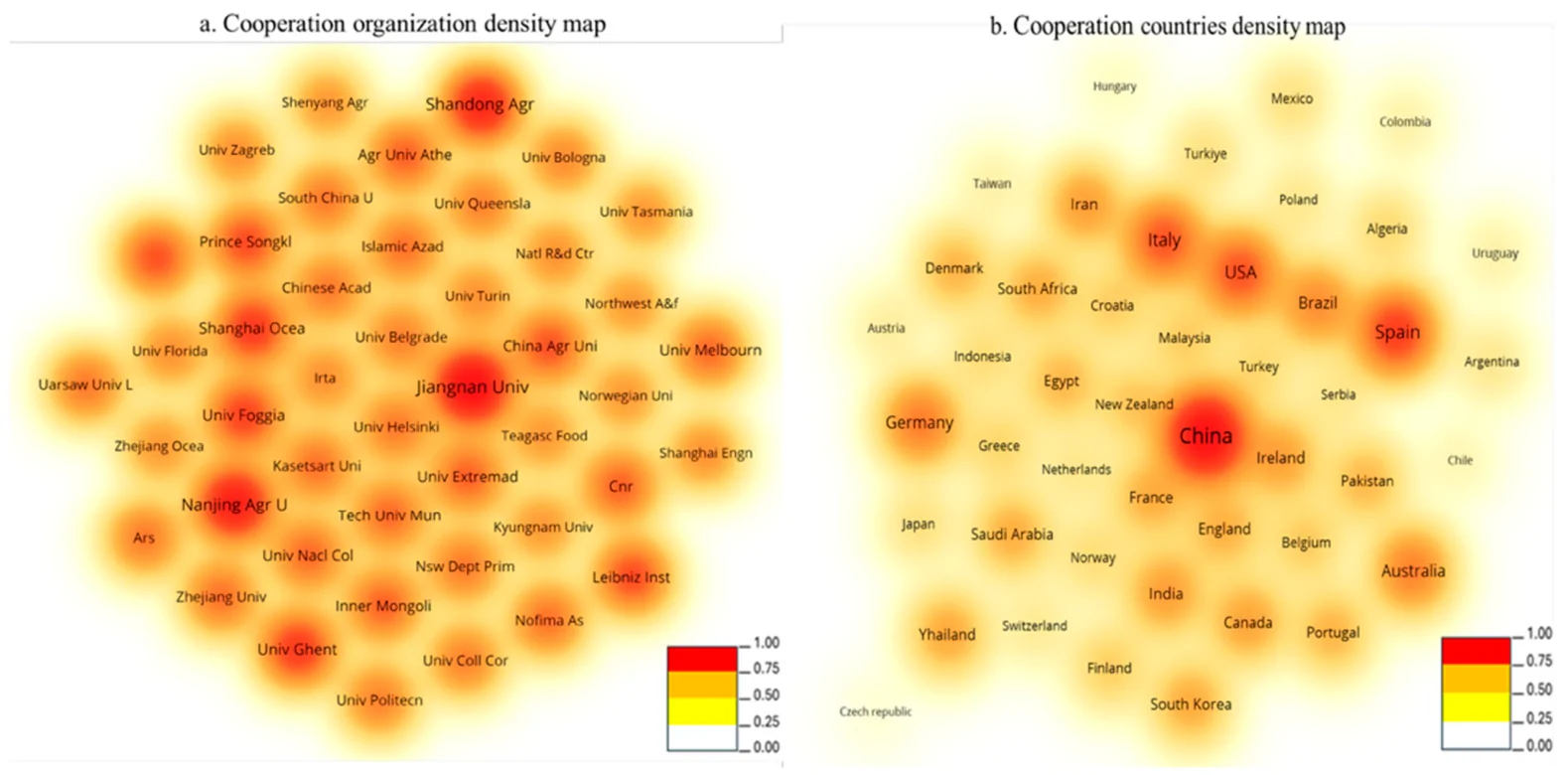
(**a**) Co-organization density map. (**b**) Co-country density map.

In the MAP field, a total of 86 countries and 1507 institutions were involved. Among them, 47 countries and 153 institutions published at least five publications, providing the basis for characterizing the collaborative network structure in the MAP field. The country and institutional collaboration networks shown in Figure 4 reflect the level of collaborative activity and network centrality within this research field. As shown in Figure 4 and Table 4, China had the highest publication output and total citation count, with 404 publications and 10,005 citations, respectively. The three institutions with the largest number of publications were Jiangnan University, Nanjing Agricultural University, and Shandong Agricultural University, with 42, 34, and 30 publications, respectively.

Chinese scholars, represented by highly productive authors such as Min Zhang, Xin Luo, and Yimin Zhang, have promoted the formation of a MAP research system centered on modified atmosphere packaging and further extended toward synergistic preservation technologies and quality regulation mechanisms. Related studies have mainly focused on the storage and preservation of fresh agricultural products and animal-derived foods, such as fruit and vegetables, meat, and aquatic products, with emphasis on the regulation of packaging gas composition, the development of active packaging materials, the application of combined preservation technologies, and the analysis of quality changes and spoilage mechanisms [62,63,64]. Based on the representative studies of highly productive authors, MAP research in China has gradually developed from the evaluation of single packaging preservation effects toward a comprehensive research direction integrating gas regulation, material innovation, multi-technology synergy, and the analysis of quality deterioration mechanisms. This trend is also consistent with the bibliometric findings in the field of active food packaging [53], indicating that China has considerable influence in active packaging research and that related studies are continuously expanding toward functional materials and food preservation applications.

Italy and Spain ranked next, with 164 and 152 publications, respectively, and total citation counts of 2998 and 3551, respectively. The core objective of MAP research in Italy and Spain is to maintain food quality, extend shelf life, and improve safety by optimizing packaging gas parameters, packaging materials, and storage conditions [72]. Related studies have gradually expanded from traditional evaluations of MAP preservation effects to intelligent monitoring, including freshness indicator labels, non-destructive detection, and shelf-life prediction [73,74], as well as sustainable packaging development, including biodegradable materials, edible coatings, and natural active packaging materials [75]. From the perspective of collaboration network distribution, Italy and Spain, as part of the European research community, have formed a strong collaborative cluster in the MAP field.

Notably, Australia published only 74 publications in the MAP field, but its total citation count reached 2889, approaching or even exceeding that of some countries with higher publication outputs. This indicates that although the scale of Australian research output is relatively limited, its findings have high academic influence and citation impact. MAP research in Australia is strongly application-oriented and closely aligned with the country’s meat and seafood industries. Representative studies have focused primarily on the microbial ecology of lamb and Atlantic salmon and on extending refrigerated shelf life, with particular attention to the effects of gas composition, packaging systems, and cold-chain conditions on product quality, microbial community dynamics, and storage stability. More recently, this research has expanded beyond passive atmosphere control toward sensor-enabled smart packaging and real-time freshness monitoring [76,77,78,79,80].

The formation of this country distribution pattern may be closely related to the development level of the domestic food industry, the intensity of research investment, the foundation of international collaboration, and the practical application demands of specific research directions. China has a large-scale agricultural production system and food industry, resulting in strong practical demand for MAP, preservation packaging, and related storage technologies. In recent years, China has continuously increased research investment in food science, packaging engineering, and agricultural product preservation. The research objects cover multiple areas, including fruit and vegetables, meat products, aquatic products, and packaging materials. This has promoted the continuous increase in the number of publications by Chinese scholars in international journals and has formed a large publication base and citation scale. In contrast, Italy and Spain, as countries with active food industries and strong food science research capacity in Europe, have a long-standing research foundation in food packaging, food safety, and storage preservation.

## 4. Research Hotspots and Theme Evolution Analysis

### 4.1. Analysis of Research Contents

**Figure 5:**
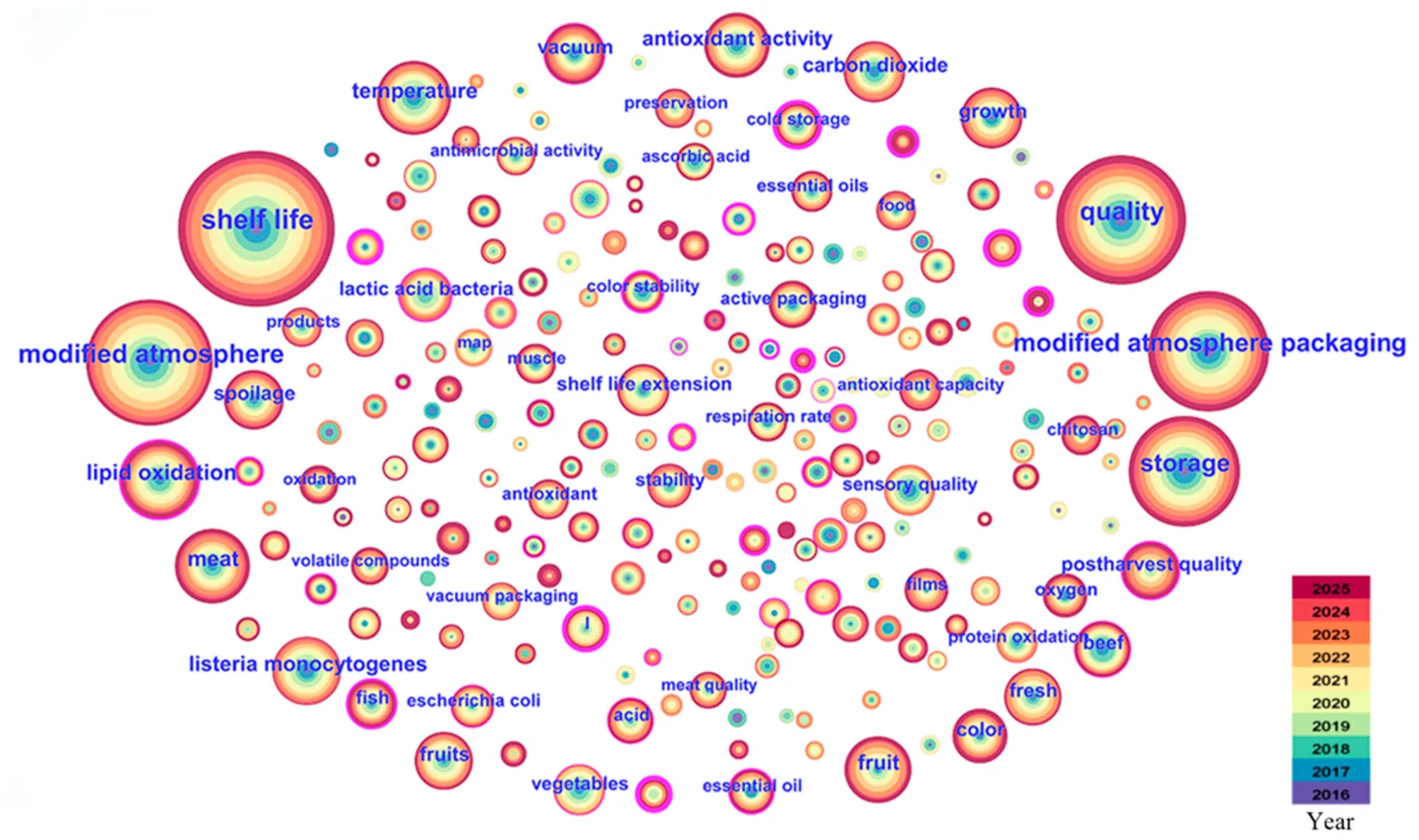
Keyword co-occurrence network map.

Keywords can effectively summarize the core content of publications, and high-frequency keywords provide an important basis for identifying research hotspots and analyzing knowledge structures [81]. Using one year as the time slice, keyword co-occurrence analysis was conducted on publications in the MAP field, and a keyword co-occurrence network map was constructed, as shown in Figure 5. In the map, nodes represent keywords; larger nodes indicate higher occurrence frequencies; links between nodes indicate co-occurrence relationships among keywords; and node colors reflect the time stage in which the keywords appeared or were active [82,83]. The keyword co-occurrence network in the MAP field contained 465 nodes and 3309 links, indicating that a relatively rich and closely connected research network has been formed in this field. In terms of the overall structure, MAP research mainly centers on core keywords such as “modified atmosphere packaging”, “storage”, “quality”, and “shelf life”, forming a research framework focused on the regulation of packaging gas parameters, maintenance of storage quality, and extension of shelf life. Among them, “shelf life” had the highest occurrence frequency, with 630 occurrences (Table 5), indicating that shelf-life extension has been one of the core research objectives in the MAP field. Keywords such as “quality”, “modified atmosphere”, “MAP”, and “storage” also showed high occurrence frequencies, further suggesting that existing studies mainly focus on maintaining food storage quality and improving preservation effects under MAP conditions. On this basis, MAP research has expanded toward quality deterioration mechanisms, microbial safety control, sensory quality maintenance, and the development of functional packaging materials. The presence of keywords such as “lipid oxidation”, “protein oxidation”, “color stability”, “sensory quality”, and “spoilage” indicates that researchers have increasingly focused on quality deterioration during MAP storage, including lipid oxidation, protein oxidation, color changes, sensory quality decline, and spoilage. These studies have attempted to analyze the dynamic changes and regulatory mechanisms of quality deterioration from a mechanistic perspective. Keywords such as “carbon dioxide”, “oxygen”, “temperature”, “vacuum”, and “cold storage” indicate that the regulation of gas composition and storage conditions remains a core approach for achieving preservation effects. Meanwhile, keywords such as “chitosan”, “essential oils”, “active packaging”, and “antimicrobial/antioxidant activity” suggest that the MAP field is gradually developing from traditional gas regulation toward active, natural, and functional approaches. These findings indicate that MAP technology is no longer limited to the simple adjustment of the packaging atmosphere, but is increasingly being combined with natural active substances, antimicrobial and antioxidant materials, and functional packaging systems, thereby promoting the expansion of food preservation research toward multifunctional synergistic preservation.

Overall, shelf-life extension and quality maintenance represent the overarching objectives of MAP research. On this basis, the research content in the MAP field can be organized into four conceptually comparable themes. (1) Atmosphere regulation and packaging system design. This theme focuses on optimizing O_2_, CO_2_, and N_2_ concentrations, storage temperature, headspace conditions, and packaging-film permeability for different food systems. In meat packaging, O_2_ helps maintain a bright-red color but may accelerate lipid and protein oxidation at high concentrations; CO_2_ is mainly used to inhibit microbial growth, whereas N_2_ generally serves as an inert filler gas. (2) Microbial ecology, safety, and spoilage control. Related studies examine total viable counts, lactic acid bacteria, specific spoilage organisms, microbial succession, and foodborne pathogens. The occurrence of keywords such as *Listeria monocytogenes* and *Escherichia coli* reflects increasing attention to microbiological safety and spoilage development under MAP conditions. (3) Physicochemical deterioration and quality regulation. This theme addresses changes in lipid and protein oxidation, pigment stability, texture, water-holding capacity, weight loss, nutritional components, and sensory quality. The specific indicators vary among meat, fruit and vegetables, and aquatic products, but collectively reveal the mechanisms through which MAP influences quality deterioration during storage. (4) Functional packaging materials and integrated preservation technologies. This theme involves the integration of MAP with chitosan films, edible coatings, essential oils, natural antioxidants, antimicrobial films, and other preservation treatments to enhance antioxidant and antimicrobial effects. Overall, these themes indicate that MAP research has gradually progressed from evaluating preservation performance toward packaging-system optimization, mechanistic analysis of microbial and physicochemical deterioration, and the development of functional and integrated preservation strategies.

Overall, the repeated occurrence of keywords such as “shelf life”, “quality”, “modified atmosphere”, “MAP”, and “storage” confirms that MAP research has long centered on core issues including food shelf-life extension, storage quality maintenance, and packaging atmosphere regulation. Keywords with high centrality play a bridging role among different research topics, reflecting their important connecting positions in the MAP knowledge network. The MAP field has formed a knowledge structure centered on shelf-life extension, quality maintenance, and gas regulation while further extending toward the analysis of quality deterioration mechanisms, the development of active packaging, and multi-technology synergistic preservation.

### 4.2. Analysis of Research Hotspots

**Figure 6:**
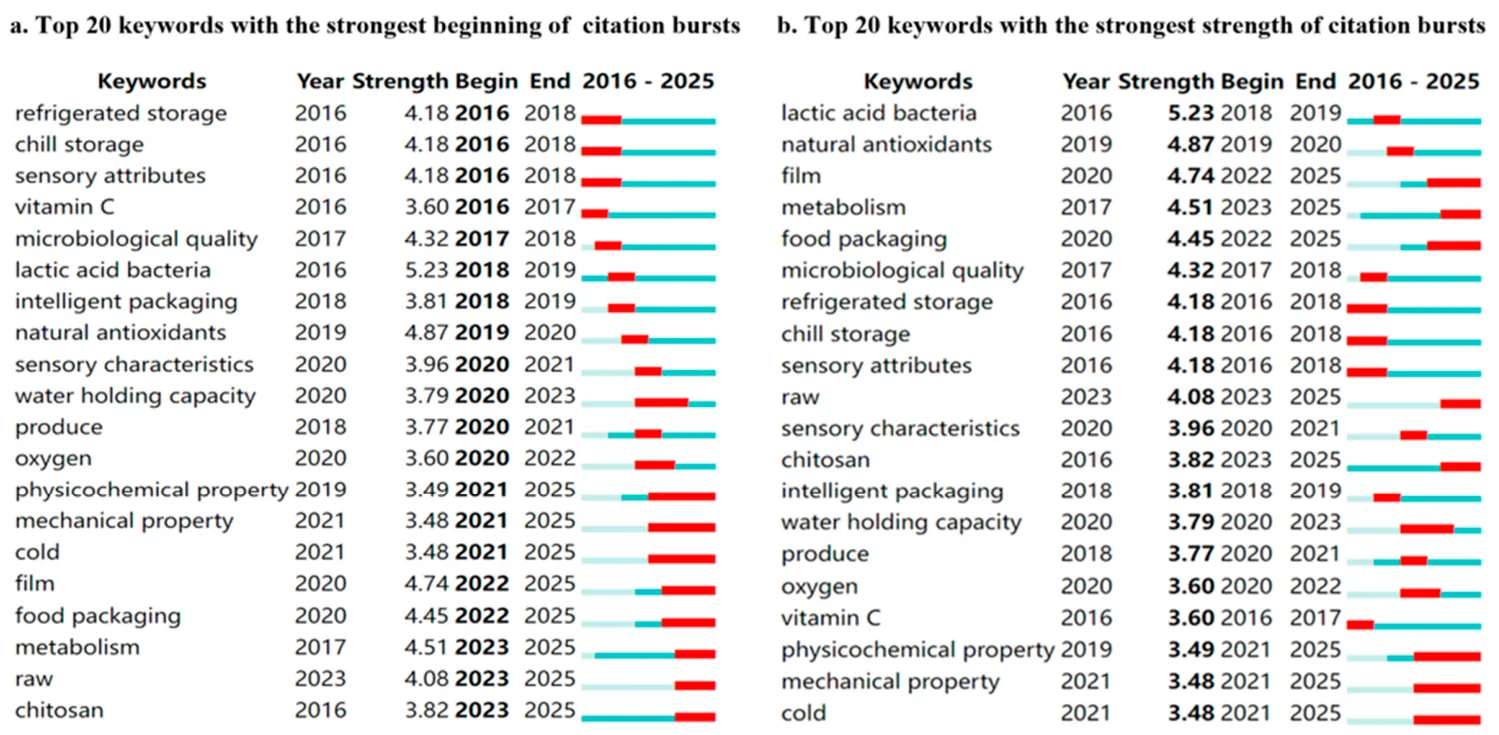
The top 20 keywords with the citation bursts: (**a**) sorted by the strength of burst, and (**b**) sorted by the beginning year of burst. Note: Year indicates the first occurrence year of the keyword; Strength represents the burst intensity; and Begin and End denote the starting and ending years of the keyword burst. Light-colored lines represent the full study period from 2016 to 2025, cyan lines indicate the years in which the keyword appeared, and red lines indicate the period during which the keyword emerged as a research frontier.

Figure 6 shows the keyword bursts and the evolution of research hotspots in the MAP field from 2016 to 2025. During 2016–2018, MAP research mainly focused on evaluating preservation performance under low-temperature storage conditions, including changes in the sensory quality, nutritional components, and microbiological quality of foods. The emergence of burst keywords such as “refrigerated storage”, “chill storage”, “sensory attributes”, “vitamin C”, and “microbiological quality” suggests increasing research attention to the combined application of MAP and temperature-controlled storage, as well as to the multidimensional assessment of food quality. In this context, “refrigerated storage” and “chill storage” represent complementary storage conditions rather than alternatives to MAP, whereas the other keywords reflect a growing emphasis on sensory, nutritional, and microbiological quality, preservation performance, and shelf-life extension. Taking meat MAP as an example, studies by Yimin Zhang [63] and Xin Luo [64] conducted relatively systematic studies on beef MAP, comparing the effects of different O_2_ and CO_2_ ratios on beef color stability, total viable counts, pH, and lipid oxidation. The experimental results showed that compared with high-oxygen MAP containing 80% O_2_ and 20% CO_2_, the three-gas MAP treatment containing 50% O_2_, 40% CO_2_, and 10% N_2_ more effectively delayed the growth of spoilage bacteria, maintained an acceptable beef color, and extended the refrigerated shelf life to 20 days [63].

From 2018 to 2021, the burst strengths of “lactic acid bacteria” (5.23) and “natural antioxidants” (4.87) were relatively high, indicating that research hotspots gradually expanded from the evaluation of single preservation effects to microbial regulation, the application of natural active substances, and combined preservation strategies. Meanwhile, the emergence of “intelligent packaging” suggests that MAP technology began to be integrated with freshness monitoring, functional packaging enhancement, and intelligent evaluation methods, promoting the development of MAP toward intelligent and multifunctional packaging. For example, the Soottawat Benjakul team combined MAP with tannic acid and green tea extract for the preservation of aquatic products, such as refrigerated seabass, striped catfish, and Pacific white shrimp [84,85]. The results showed that the treatment combining tannic acid with MAP had the lowest peroxide value thiobarbituric acid reactive substances (TBARS), and non-heme iron content, and extended the shelf life to 15 days, which was five times that of the air-packaged control group. The combination of green tea extract and MAP further slowed the increases in pH and total volatile basic nitrogen (TVB-N) and inhibited chemical deterioration. The team’s research promoted the development of MAP from single gas regulation toward the application of natural antioxidants and combined preservation strategies. In addition, Jing Xie and colleagues applied slightly acidic electrolyzed water combined with MAP technology to aquatic products such as farmed pufferfish [86]. The results showed that slightly acidic electrolyzed water combined with MAP, especially the gas mixture of 60% CO_2_, 5% O_2_, and 35% N_2_, significantly inhibited the growth of spoilage microorganisms, reduced the levels of total volatile basic nitrogen (TVB-N), trimethylamine, and thiobarbituric acid reactive substances (TBARS), and extended the refrigerated shelf life of pufferfish from approximately 8 days to 18 days. This expanded the research perspective on the synergistic preservation of MAP combined with non-thermal processing technologies.

Since 2021, research hotspots in the MAP field have further shifted toward the functionalization of packaging materials, optimization of packaging performance, and analysis of internal mechanisms. Keywords such as “film” (4.74), “food packaging” (4.45), and “chitosan” (3.82) showed relatively high burst strengths and continued until 2025, indicating that packaging films, food packaging systems, and natural biodegradable materials have become research hotspots. In addition, the application of natural materials such as chitosan has strengthened the focus of MAP research on sustainable, safe, and functional packaging materials. The burst of “metabolism” suggests that researchers have begun to explore the regulatory mechanisms of MAP in maintaining food quality and delaying deterioration from the perspective of metabolic changes, promoting the extension of this field from apparent quality evaluation to internal mechanism analysis. Qu et al. [62] systematically summarized the application of microporous-film MAP in fresh food preservation and pointed out that by regulating the gas permeability of film materials, micropore size, pore density, and packaging structure, the O_2_ and CO_2_ concentrations inside the package can be matched with the respiratory metabolism of foods, thereby improving the regulatory precision and preservation performance of the packaging system. Jorgen Lerfall, Bjorn Tore Rotabakk, and colleagues [87] combined soluble gas stabilization technology with MAP. By pre-dissolving CO_2_ into the food matrix, soluble gas stabilization improved CO_2_ utilization efficiency and integrated this process into the cooling stage of surimi products, reflecting the expansion of MAP research from gas composition regulation toward packaging system optimization and integration with processing procedures.

In conclusion, from 2016 to 2025, the MAP field underwent a transition from low-temperature storage and quality evaluation to combined preservation, natural active substances, intelligent packaging, functionalized packaging materials, and the analysis of quality change mechanisms. Current research hotspots mainly focus on the development of packaging films, the application of natural biodegradable materials, the optimization of food packaging performance, and the mechanisms underlying quality and metabolic changes during storage. This indicates that MAP research is gradually shifting from traditional preservation effect evaluation toward multi-technology integration, functional packaging development, and mechanistic studies.

### 4.3. Analysis of Research Trends

**Figure 7:**
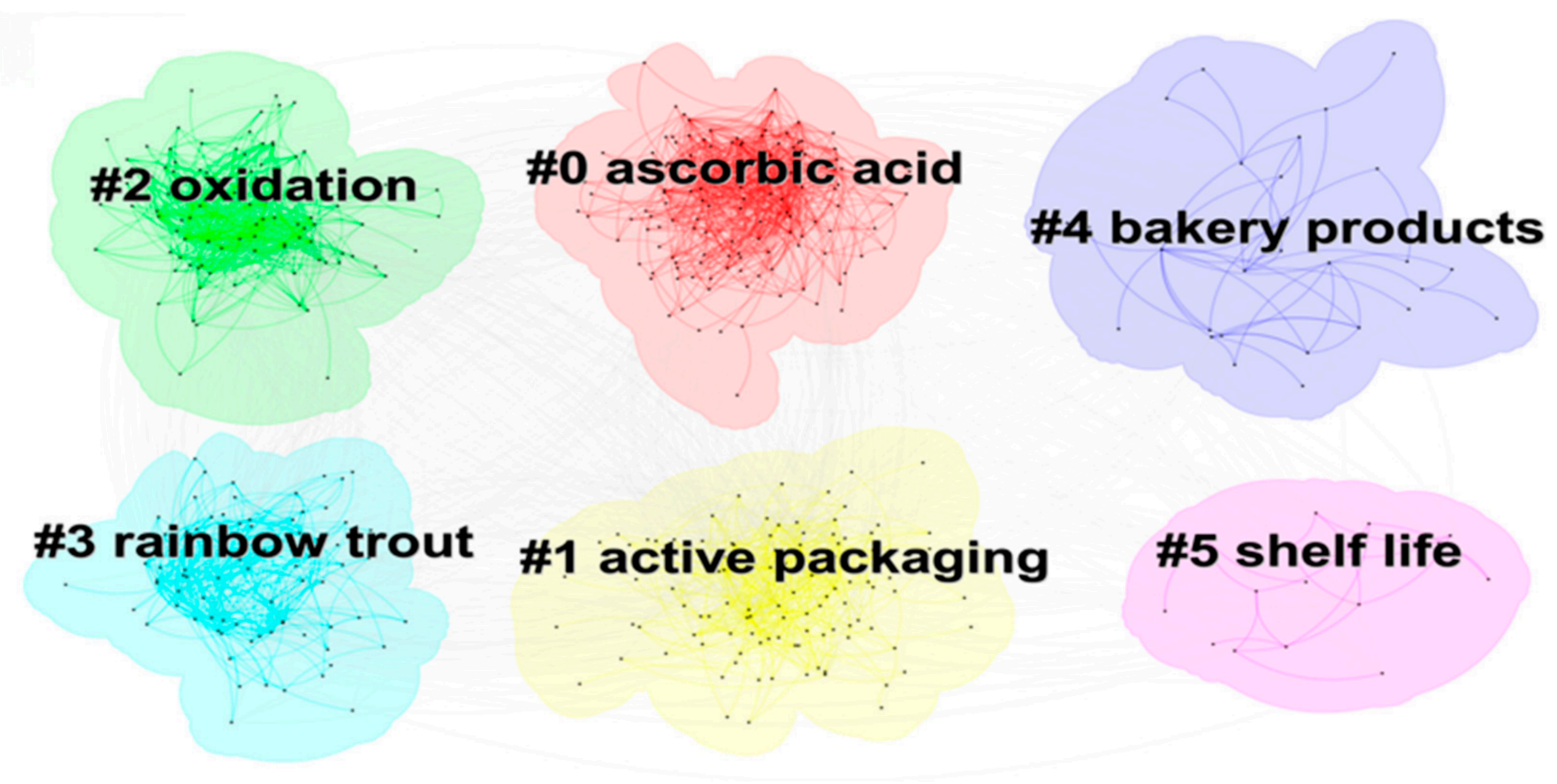
The network of keywords cluster in MAP.

To identify research trends and thematic evolution characteristics in the MAP field, thematic clustering analysis was performed on the keyword co-occurrence network using the Pathfinder network pruning method. As shown in Figure 7, based on the co-occurrence relationships and link strengths among keywords, the keyword network was divided into six major clusters: #0 ascorbic acid, #1 active packaging, #2 oxidation, #3 rainbow trout, #4 bakery products, and #5 shelf life. The clustering evaluation results showed that the modularity value Q of the keyword clusters in the WOSCC dataset was 0.3693 (>0.3), and the mean silhouette value S was 0.7022 (>0.7), indicating that the clustering results were reasonable and had good clustering quality [82,88,89].

In terms of cluster content, cluster #0 ascorbic acid is located at the center of the network and is closely connected with several other clusters, indicating a strong hub role within the research network. This cluster mainly reflects research topics related to antioxidant activity, quality maintenance, and the stability of nutritional components. Ascorbic acid is a water-soluble vitamin and natural reducing agent widely present in fruits, vegetables, and their processed products. It can scavenge reactive oxygen species, retard lipid and pigment oxidation, and inhibit enzymatic browning by reducing quinone intermediates. Accordingly, in MAP preservation studies, ascorbic acid can be used as an exogenous antioxidant treatment to alleviate browning and quality deterioration in fruits and vegetables. For example, Li et al. [90] applied ascorbic acid in combination with high-oxygen MAP to preserve fresh-cut eggplant. In addition, its endogenous content or retention rate is commonly monitored as an indicator of changes in nutritional quality during processing and storage, as demonstrated by Sthapit Kandel et al. [91], who evaluated ascorbic acid content and retention in MAP-stored fresh-cut lettuce. Clusters #1 active packaging and #2 oxidation were closely associated with the central cluster, suggesting that the development of active packaging and the control of oxidative deterioration are important research directions in the MAP field. Clusters #3 rainbow trout and #4 bakery products corresponded to the application of MAP in aquatic products and bakery products, respectively, reflecting the diversified and product-specific development of MAP research across different food systems. Cluster #5 shelf life represented shelf-life extension, which is a core objective throughout MAP-related studies. Overall, the MAP field has formed a relatively stable thematic structure centered on quality maintenance and oxidation control, extended by the development of active packaging, and supported by applications in typical food systems.

**Figure 8:**
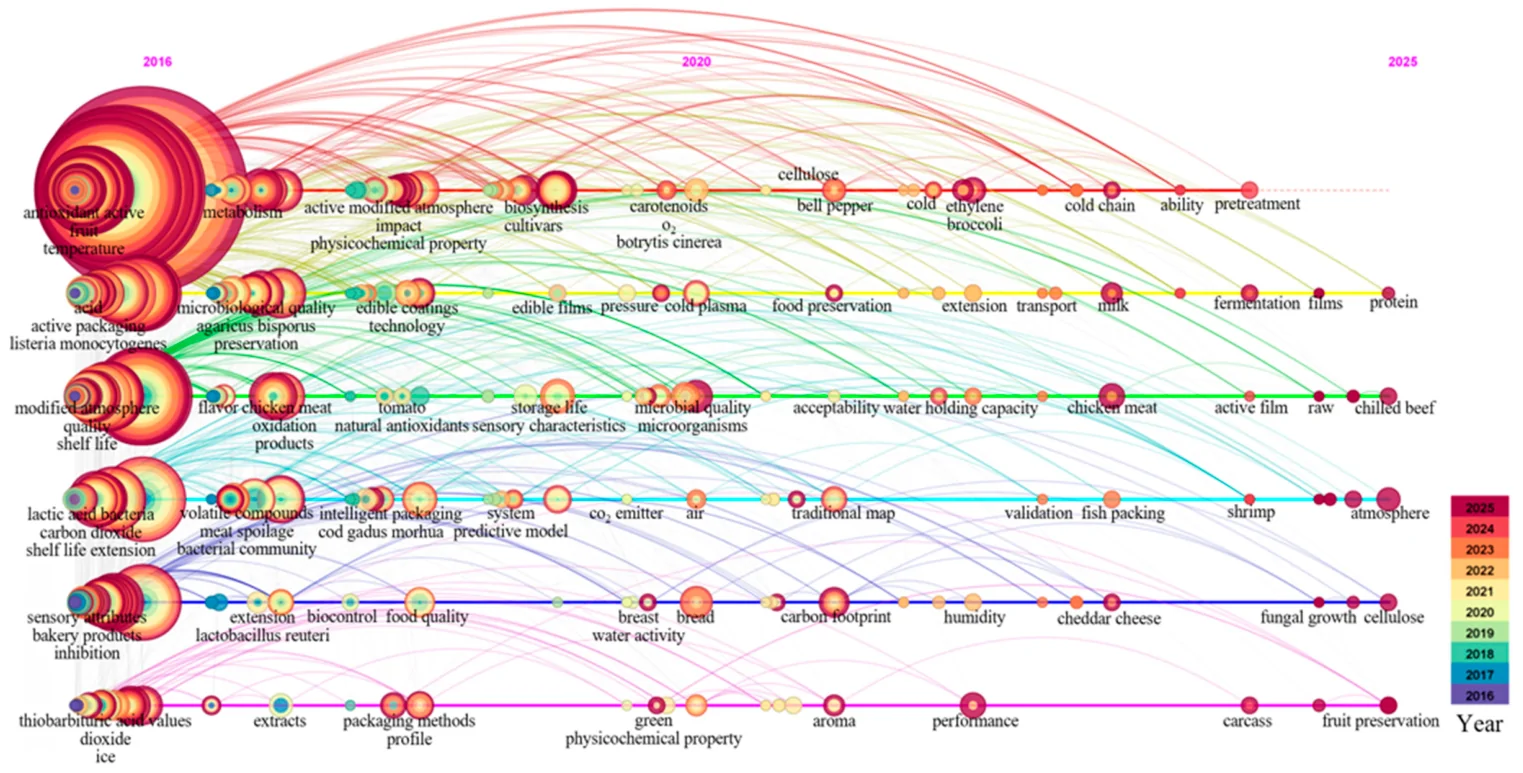
The keywords time zone view in 2016–2025.

From the timeline perspective (Figure 8), research hotspots in the MAP field show a staged evolutionary pattern, shifting from the verification of application effects to integrated preservation strategies, and further toward mechanistic analysis and sustainable functional development. During 2016–2018, research mainly focused on the basic preservation effects of MAP, with emphasis on shelf-life extension, spoilage control, microbiological quality, and sensory quality maintenance. These studies primarily aimed to verify the preservation performance of MAP in the storage of perishable foods.

During 2018–2021, the research focus gradually shifted from the evaluation of macroscopic preservation effects to the regulation of quality deterioration processes. Issues such as oxidative reactions, microbial safety, moisture retention, and texture changes received increasing attention. MAP also began to be combined with technologies such as edible coatings, natural antioxidants, and cold plasma, forming multi-technology synergistic preservation strategies. During 2021–2025, MAP research further expanded toward the functionalization of packaging materials, utilization of natural active substances, and analysis of quality change mechanisms. Topics such as active packaging, plant-derived functional substances, and high-value utilization of agricultural by-products remained active, reflecting the trend toward sustainable and functional development in this field. Meanwhile, the emergence of keywords such as metabolism, volatile organic compounds, quality prediction models, and carbon footprint indicates that researchers have begun to deepen their understanding of MAP mechanisms from the perspectives of intrinsic metabolic responses, identification of flavor markers, quality prediction, and environmental impact assessment.

## 5. Highly Cited Literature and Intellectual Base Analysis

Highly cited publications play important structural roles in the local citation network and can collectively reflect the core knowledge base and major developmental milestones of the MAP field [82,92,93]. To further reveal the knowledge foundation of the MAP field, this study focused on highly cited core publications through chronological citation mapping, citation cluster analysis, and main path analysis to obtain more detailed insights into knowledge evolution [94].

### 5.1. Analysis of Citation Chronology Maps

**Figure 9:**
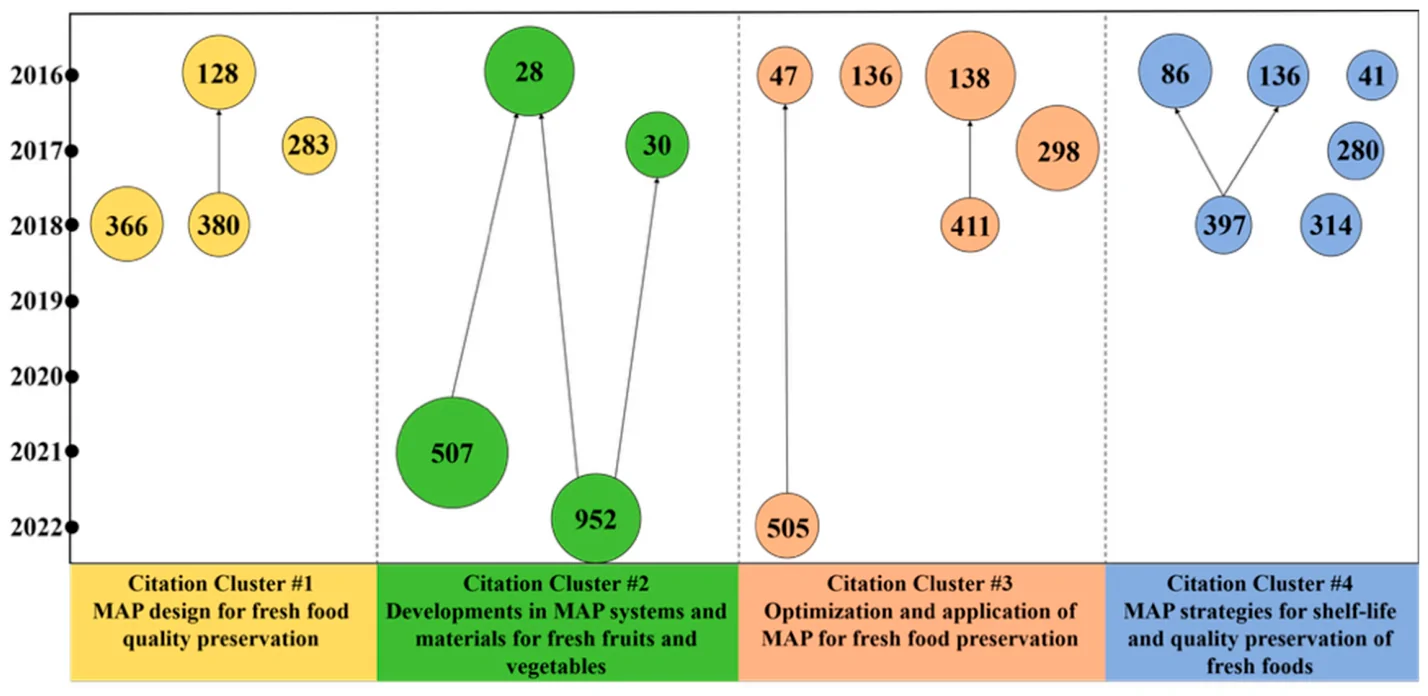
Citation chronology and topic Cluster map in MAP.

A chronological citation map is a citation network of key publications arranged along a time axis and constructed based on citation relationships among documents. It can visually present the internal connections among core publications and the pathways of knowledge inheritance [95]. The specific procedure was as follows: HistCite was used to arrange the 1568 publications in chronological order according to publication year and assign document codes ranging from 1 to 1568. Using the Graph Maker function, the settings “Select by = LCS” and “Limit = 20” were applied to select the 20 publications with the highest local citation counts for in-depth analysis, and a chronological citation map was then generated. The number of selected publications was determined by comprehensively considering 1% of the total analysis sample and a practical rule of using multiples of 5 or 10 [96]. Detailed information on these publications is presented in Table 6. As shown in Figure 9, nodes aligned with the years represent publications published in the corresponding year. The numbers inside the nodes indicate document codes, and the radius of each node is proportional to its LCS value. The arrows between nodes represent citation relationships among publications, with the arrowhead pointing to the cited publication and the arrow tail pointing to the citing publication. The important indicators involved in the analysis include the LCS, which refers to the citation count of a publication within the local database, namely the 1568-publication sample in this study, and the GCS, which refers to the total number of citations received by a publication in a specific database, namely the Web of Science database in this study.

As shown in Figure 9, the 20 selected highly cited publications were distributed between 2016 and 2022, and clear citation links were observed among them, indicating that this period represented an important stage of knowledge accumulation and rapid evolution in the MAP field. From 2016 to 2018, MAP research entered a stage in which the core knowledge base was rapidly consolidated, with a total of 17 core nodes appearing. The research content mainly focused on the preservation effects of MAP in different fresh foods, with particular attention to fundamental issues such as shelf-life extension, quality maintenance, and spoilage inhibition. Meanwhile, citation links among publications gradually strengthened, and the research perspective shifted from the simple evaluation of preservation effects toward the improvement of packaging materials, the design of microperforated films, the regulation of headspace atmosphere, and the optimization of equilibrium modified atmosphere packaging (EMAP) systems. From 2020 to 2022, MAP research further showed characteristics of integrative development. Although the number of core nodes decreased, thematic integration among highly influential publications became more prominent. This indicates that research at this stage placed greater emphasis on the comprehensive use and further extension of previous findings, with the research focus gradually expanding to deeper issues such as microbial community succession, mechanisms of volatile metabolite formation, dynamic modeling, and intelligent monitoring. However, the number of newly emerging core publications in recent years has been relatively limited, which may be related to insufficient time for citation accumulation and the lag effect of citations. Overall, the knowledge base of the MAP field has evolved from the verification of application effects to packaging system optimization, and further toward mechanism analysis and intelligent expansion. This evolutionary pattern is generally consistent with the conclusions drawn from the above analyses of publication trends and keyword evolution.

### 5.2. Analysis of Citation Cluster

The node positions and link relationships in the chronological citation map show certain clustering characteristics. To further identify the structural associations and research topics among core publications in the MAP field, Pajek 6.02 software was used to divide the top 20 publications ranked by Local Citation Score (LCS) into four citation communities with relatively close thematic connections [97]. As shown in Figure 9, the yellow, green, orange, and blue regions represent citation communities 1–4, respectively. Citation cluster analysis focuses more on citation relationships and knowledge inheritance pathways among core publications, revealing more fine-grained research content and thematic structures in the MAP field. The specific contents of the four citation communities are as follows:

Citation cluster 1 was characterized as “Design studies of MAP for quality maintenance in fresh foods”. This cluster mainly focuses on headspace atmosphere regulation, volatile organic compound/off-odor formation, and quality responses in MAP systems, with research objects including fresh products such as broccoli, cod, strawberries, and Tricholoma matsutake. Related studies primarily examined the effects of packaging material permeability to gases and water vapor, microperforation design, headspace humidity, and gas composition regulation on storage quality and sensory deterioration. Among these studies, Caleb [98] and Wei [99] achieved the coordinated optimization of gas composition and humidity inside packages through cellulose windows, microperforation, and functional packaging design, thereby reducing condensation and the accumulation of undesirable volatiles. Kuuliala [100] linked volatile organic compounds (VOCs) with microbial spoilage, sensory rejection, and intelligent packaging warnings, promoting the extension of MAP research from preservation effect evaluation toward the analysis of volatile metabolic mechanisms and the development of spoilage indicator technologies. Overall, this community reflects the trend of MAP research shifting from packaging material design and quality maintenance toward precise headspace regulation, off-odor control, and intelligent monitoring.

Citation cluster 2 can be summarized as “Research progress in MAP systems and packaging materials for fresh fruit and vegetables”. This cluster community mainly focuses on the improvement of packaging materials, regulation of film structures, and coordinated optimization of the gas and humidity environment inside packages. Related publications indicate that research on MAP for fruit and vegetables has undergone a continuous transition from traditional preservation applications toward more precise packaging design. Wilson [26] highlighted the application value of MAP in the preservation of whole and fresh-cut fruit and vegetables, emphasizing its role in delaying respiratory metabolism, maintaining quality, and extending shelf life. Mistriotis [76] extended this research to equilibrium modified atmosphere packaging (EMAP) systems and the development of biodegradable materials, reflecting a trend that emphasizes both preservation performance and environmental sustainability. Qu [62] pointed out that microporous-film MAP can improve the adaptability and controllability of packaging systems by regulating film permeability to match the respiration characteristics of fruit and vegetables. Jalali [101] further introduced humidity regulation and mathematical simulation methods, promoting the optimization of the packaging environment from empirical design toward model-based regulation. Overall, this cluster reflects the development trend of MAP research for fruit and vegetables toward sustainability, precision, and model-based design.

Citation cluster 3 can be summarized as “Optimization and application of MAP in fresh food preservation”. This cluster mainly focuses on MAP system design and its role in quality regulation in fresh foods. Early studies emphasized the construction of mathematical models for MAP in fruit and vegetables. By integrating factors such as product respiration rate, packaging material permeability, and storage temperature, these studies simulated the dynamic changes in O_2_ and CO_2_ concentrations inside packages, highlighting the importance of coordinated optimization among product physiological characteristics, packaging material properties, and storage environment for MAP design [102]. Subsequent studies by Matar [103], Gholami [104], Rodrigues [105], and others applied MAP to various fresh foods, including strawberries, mushrooms, aquatic products, and meat products, focusing on evaluating the effects of different gas compositions and packaging conditions on shelf-life extension and quality maintenance. Related studies have expanded from the evaluation of preservation effects to microbial control, oxidative stability, moisture retention, and quality deterioration mechanisms [106]. This reflects the developmental trend of MAP research from theoretical model construction to multi-product application, and further toward more refined quality regulation.

Citation cluster 4 can be summarized as “MAP strategies for shelf-life extension and quality preservation of fresh foods”. This cluster table mainly focuses on the preservation effects and underlying mechanisms of MAP in meat, fruit, and vegetables [37,107]. Early studies mainly compared the effects of different gas compositions on the shelf life, color stability, oxidative reactions, and spoilage microbiota of products such as beef and chicken. For example, Yang [63] found that although high-oxygen MAP helped maintain the bright red color of beef in the short-term, it could accelerate oxidation and limit shelf life, whereas anaerobic MAP or CO-MAP performed better in inhibiting oxidation, reducing microbial counts, and maintaining color stability. Subsequently, research further advanced toward the analysis of spoilage microbiota succession and metabolic changes. Yang [63] and Höll [108] used high-throughput sequencing and other approaches to reveal the succession patterns of dominant spoilage bacteria under different MAP systems and their relationships with physicochemical deterioration, indicating that this field has gradually shifted from preservation-effect evaluation to micro-level mechanistic analysis. In the field of fruit and vegetables, research has also expanded from preservation technologies for fresh-cut products to mathematical modeling of dynamic gas changes inside packages [109]. Overall, this cluster reflects the continuous deepening of MAP research from the evaluation of application effects toward spoilage mechanism analysis and predictive modeling.

Taken together, the four citation communities indicate that the core literature in the MAP field mainly focuses on packaging system design, the optimization of preservation packaging systems for fruit and vegetables, quality regulation of fresh foods, and shelf-life extension and spoilage mechanism analysis. Although these communities differ in terms of research objects and technical approaches, the overall pattern collectively reflects the developmental trend of MAP research from early preservation-effect evaluation toward precise packaging system design, analysis of quality deterioration mechanisms, and model-based prediction. Meanwhile, the emergence of topics such as active packaging, microperforated films, humidity regulation, volatile metabolite identification, and microbial community analysis also suggests that the field is expanding from single gas regulation toward multi-factor synergistic regulation, as well as intelligent and mechanistic research.

## 6. Research Challenges and Future Trends

The combined evidence from publication performance, collaboration networks, keyword analysis, and citation structures indicates that the MAP field is undergoing not merely quantitative growth, but a broader structural transition. Three notable asymmetries can be identified: publication output does not fully correspond to citation influence; frequently studied outcomes are not necessarily the concepts that connect different knowledge domains; and rapidly emerging technological topics have not yet developed into a fully consolidated intellectual base. These asymmetries reveal several challenges that cannot be identified by examining individual bibliometric indicators separately.

### 6.1. Research Collaboration and Regional Differences

MAP research has attracted broad international participation, but the distribution of knowledge production and academic influence remains uneven. China produced the largest number of publications and accumulated the highest total number of citations, whereas Australia generated substantially fewer publications but achieved a comparatively high citation impact relative to its publication volume. Although such unnormalized citation comparisons should be interpreted cautiously because of differences in publication year and research composition, they suggest that research scale and academic influence do not necessarily increase in parallel.

A similar pattern was evident in the author collaboration network. Although 126 authors published at least five papers, only 47 authors formed clear collaborative relationships within the thresholded core network. This indicates that MAP research has developed several productive research groups, but knowledge exchange among these groups remains limited. Country- and commodity-specific specialization has contributed to advances in fruit and vegetable physiology, meat oxidation, seafood spoilage, packaging materials, and intelligent monitoring; however, it has also resulted in partially separated knowledge communities.

Future research should therefore move beyond isolated institutional or commodity-specific studies toward international and interdisciplinary research consortia. Shared experimental protocols, interoperable datasets, and multicenter validation should be established to compare packaging systems across products, climates, distribution conditions, and regulatory environments. Such collaboration would help distinguish general principles of MAP design from effects that are specific to individual commodities or experimental settings.

### 6.2. From Preservation Effects to Mechanism Analysis

A comparison of keyword frequency and network centrality reveals an important structural characteristic of the MAP knowledge system. “Shelf life”, “quality”, “modified atmosphere”, and “storage” were among the most frequent keywords, but their centrality values were relatively low. In contrast, “lipid oxidation” occurred less frequently but showed the highest centrality, indicating a strong bridging role among different research themes. This frequency–centrality mismatch suggests that shelf-life extension and quality maintenance remain the dominant stated objectives of MAP research, whereas the intellectual integration of the field is increasingly driven by specific deterioration mechanisms and packaging-related processes.

This finding also exposes several unresolved trade-offs. For example, high-O_2_ atmospheres can maintain the desirable red color of meat but may accelerate lipid and protein oxidation. Increased CO_2_ concentrations can inhibit microbial growth, but inappropriate atmospheres may induce off-odors, physiological disorders, or anaerobic metabolism in some products. Similarly, an extension of sensory acceptability does not necessarily guarantee a corresponding extension of microbiological safety. Consequently, optimizing MAP according to a single outcome, such as color, microbial count, or nominal shelf life, may shift deterioration from one pathway to another rather than achieve an overall improvement.

Future MAP studies should adopt multi-objective optimization frameworks that simultaneously consider microbial safety, oxidative stability, sensory quality, nutritional retention, package performance, environmental impact, and economic feasibility. Rather than reporting only the best-performing gas ratio, researchers should identify trade-off surfaces and acceptable operating windows for different products. This transition would shift MAP design from single-response optimization toward mechanism-based decision making.

### 6.3. Development of Active, Intelligent, and Sustainable MAP

Keyword burst and timeline analyses show that films, food packaging, chitosan, metabolism, physicochemical properties, and mechanical properties have remained active research fronts in recent years. These results indicate a clear expansion toward bio-based materials, functional films, active packaging, and mechanism-oriented quality analysis. However, the highly cited local knowledge base remains concentrated mainly in publications from 2016–2022, and the major citation communities are still dominated by conventional topics such as fresh-food packaging design, gas and humidity regulation, shelf-life extension, and commodity-specific quality preservation.

This discrepancy suggests that active, intelligent, and sustainable MAP technologies are expanding research fronts but have not yet become fully consolidated technological systems. The pattern may partly reflect citation-window effects, because recent studies have had less time to accumulate citations. Nevertheless, it also indicates that many emerging systems remain at the laboratory or proof-of-concept stage.

Future studies should therefore evaluate functional packaging materials through a complete performance framework rather than through antimicrobial or antioxidant activity alone. Barrier properties, mechanical stability, active-agent release kinetics, food-contact safety, processing compatibility, recyclability or biodegradability, cost, and sensory effects should be examined simultaneously. Pilot-scale production and validation under actual storage and distribution conditions are also required before emerging materials can be regarded as industrially viable MAP solutions.

### 6.4. Dynamic Design and Predictive Modeling of MAP Systems

MAP is intrinsically a dynamic system in which package atmosphere changes through interactions among food respiration or metabolism, gas transfer through packaging materials, temperature and humidity fluctuations, microbial activity, and physicochemical deterioration. Citation clusters related to gas-exchange modeling, microperforated films, humidity regulation, volatile metabolites, and microbial succession demonstrate increasing recognition of this dynamic nature. However, many studies still rely on fixed initial gas compositions, constant storage temperatures, and end-point quality measurements.

This methodological mismatch limits the transferability of experimental conclusions to real supply chains, where temperature abuse, package leakage, product variability, and changes in respiration or microbial activity frequently occur. In addition, intelligent packaging research has focused more strongly on sensing and visual indication than on integrating sensing outputs with predictive models and adaptive packaging control.

Future research should couple mass-transfer models with commodity respiration, microbial growth, oxidation kinetics, moisture migration, and sensory deterioration. Physics-based models may be integrated with machine learning and real-time sensor data to update shelf-life predictions during distribution. However, model performance should be evaluated using independent products, production batches, temperature profiles, and packaging configurations rather than only through goodness-of-fit within a single experiment. The long-term objective should be to progress from passive freshness indication toward decision-support and adaptive MAP management.

### 6.5. Standardization and Industrial Validation

The keyword clusters and citation communities combine mechanism-related topics, such as oxidation and active packaging, with commodity-specific topics, such as rainbow trout, bakery products, fruit and vegetables, meat, and aquatic products. This mixed structure reflects an inherent characteristic of MAP: its effectiveness is strongly dependent on product physiology, composition, microbial ecology, and distribution conditions. However, it also reveals that knowledge remains fragmented across commodity groups.

Differences in experimental design further restrict comparison among studies. Gas composition may be reported only as the initial nominal ratio, without adequate information on package volume, product-to-headspace ratio, film permeability, package geometry, actual atmosphere evolution, temperature history, or humidity. Shelf-life endpoints and microbiological acceptability criteria also vary considerably among products and studies. These inconsistencies make it difficult to determine whether conflicting results arise from genuine commodity differences or from methodological variation.

Future research should develop minimum reporting standards for MAP experiments, including packaging configuration, material properties, headspace conditions, gas evolution, storage history, microbiological criteria, physicochemical indicators, and sensory rejection thresholds. Cross-commodity studies should not seek a universal gas composition; rather, they should identify transferable design principles and clearly define the boundaries within which individual models are valid. Industrial validation should include realistic cold-chain fluctuations, packaging-line compatibility, economic analysis, food-contact safety, and consumer acceptance.

## 7. Conclusions

This study conducted a bibliometric and visualization analysis of MAP research in the field of food and agricultural products from 2016 to 2025. The results showed that MAP research maintained a high level of activity over the past decade and has gradually expanded from application-oriented studies focusing mainly on shelf-life extension and quality maintenance into an interdisciplinary research field involving packaging materials, microbial safety, quality regulation, mechanistic analysis, active packaging, and intelligent monitoring. A broad international participation network has been formed in the MAP field. Countries such as China, Italy, Spain, the United States, and Australia have shown outstanding performance in terms of publication output and academic influence, and have established a solid research foundation around perishable foods such as fruit and vegetables, meat products, and aquatic products. However, cross-regional, cross-institutional, and interdisciplinary collaboration still needs to be further strengthened.

Current research hotspots in the MAP field have gradually shifted toward active packaging, intelligent packaging, bio-based materials, natural functional components, volatile organic compounds, microbial community succession, and mechanisms of quality deterioration. This transition from application-oriented validation to system design and mechanistic analysis indicates that MAP technology is developing toward greater precision, sustainability, functionality, and model-assisted optimization. The present study may provide a useful reference for future topic selection, packaging-system design, and the development of food-preservation technologies in the MAP field. Future research should further investigate the coupled relationships among food characteristics, packaging-material properties, gas transfer, microbial dynamics, and quality deterioration, while also conducting dynamic modeling, multi-objective optimization, intelligent monitoring, and industrial-scale validation under realistic storage and distribution conditions.

The results of this study are based on the analysis of objective data, but there is a limitation associated with this study. In order to meet the data analysis requirements, we only used literature originally published in English from the WOSCC database. Therefore, the inclusion of papers published in other languages from other databases may increase the rigor of the study. Some studies employing alternative terminology may not have been retrieved, even though their technical content may be relevant to MAP. Future studies looking at a greater number of databases (such as Scopus and field-specific literature databases) and different terminology (such as modified atmosphere storage and controlled atmosphere packaging) with broader coverage are strongly encouraged.

## Figures and Tables

**Table 1:** Literature search formula and search results.

Set	Search Formula	Number of Valid Literatures
#1	TS = (“modified atmosphere packaging”)	4570
#2	(#1) and DT = (“article” or “review article”)	4512
#3	(#1) and (#2) and PY = (2016 or 2017 or 2018 or 2019 or 2020 or 2021 or 2022 or 2023 or 2024 or 2025)	2374
#4	(#1) and (#2) and (#3) and WC = (Food Science Technology)	1580

Source: Compiled by the authors.

**Table 2:** Top 10 collaborators in MAP.

Rank	Co-Author	Research Focus	Documents	Citations
1	Yimin Zhang	Beef MAP and shelf-life quality	29	693
2	Min Zhang	Fresh fruit and vegetables MAP preservation	27	1450
3	Xin Luo	Beef MAP focusing on color stability, spoilage control and shelf life	25	694
4	Lixian Zhu	Meat quality and storage stability under MAP	21	672
5	Jing Xie	MAP and combined preservation technologies for aquatic products	19	559
6	Soottawat Benjakul	Seafood quality deterioration and shelf-life extension	18	491
7	Rongrong Liang	Beef color and oxidation stability	17	422
8	Xiaoyin Yang	Quality changes and microbial dynamics of beef steaks under MAP	16	431
9	Jorgen Lerfall	Salmon MAP and quality stability during storage	16	201
10	Bjorn tore Rotabakk	Seafood MAP and packaging system design	16	182

Source: Compiled by the authors.

**Table 3:** Top 10 co-cited authors in MAP.

Rank	Co-Cited Author	Research Focus	Citations
1	Adel A. Kader	Physiological basis and design principles of MAP for fresh produce	220
2	Morten Sivertsvik	MAP of fish and seafood: microbial growth, gas composition	191
3	Oluwafemi James Caleb	Fresh produce MAP, active packaging, quality monitoring	151
4	Lone Gram	Spoilage microbiota and specific spoilage organisms in MAP seafood	146
5	Paw Dalgaard	Predictive microbiology and shelf-life modeling of MAP seafoods	146
6	Shukadev Mangaraj	MAP package design, gas exchange and modeling	138
7	Kenneth W. McMillin	Meat MAP, high-O_2_/CO-MAP	136
8	Danilo Ercolini	Microbial community succession in MAP foods	124
9	Pramod V. Mahajan	MAP modeling, respiration kinetics	121
10	Marianne N. Lund	Lipid/protein oxidation and quality deterioration in MAP meat	117

Source: Compiled by the authors.

**Table 4:** Top 10 countries and top 10 organizations in MAP publications.

Country	Documents	Citations	Organization	Documents	Citations
China	404	10,005	Jiangnan University	42	1853
Italy	164	2998	Nanjing Agricultural University	34	947
Spain	152	3551	Shandong Agricultural University	30	728
USA	101	2136	Shanghai Ocean University	24	603
Australia	74	2889	Ghent University	24	595
Germany	72	1789	Leibniz Inst. ATB	23	435
India	70	2057	University of Foggia	22	301
Iran	67	2038	Prince of Songkla University	20	512
South Korea	57	1426	China Agricultural University	19	403
Brazil	53	1848	University of Zaragoza	19	420

Source: Compiled by the authors.

**Table 5:** Top 20 keywords in MAP research.

Rank	Keyword	Freq	Centrality	Rank	Keyword	Freq	Centrality
1	Shelf life	630	0.02	1	Antioxidant activity	111	0.09
2	Quality	425	0.01	2	Carbon dioxide	103	0.05
3	Modified atmosphere	411	0	3	Growth	100	0.03
4	MAP	377	0.03	4	Vacuum	98	0.11
5	Storage	322	0	5	Spoilage	97	0.03
6	Lipid oxidation	162	0.53	6	Fruits	97	0.04
7	Meat	156	0	7	Postharvest quality	90	0.22
8	Temperature	148	0.02	8	Fresh	89	0
9	*Listeria monocytogenes*	126	0.03	9	Color	85	0
10	Fruit	122	0.03	10	Beef	82	0.12

Source: Compiled by the authors.

**Table 6:** The list of the 20 most cited publications.

Code	LCS	GCS	Reference
507	40	153	Matthew Deas Wilson et al. (2019)
952	33	160	Ping Qu et al. (2022)
138	33	81	Zinash A. Belay et al. (2016)
28	33	78	Mistriotis, Antonis et al. (2016)
298	31	127	Rashid Gholami et al. (2017)
366	27	95	Lotta Kuuliala et al. (2018)
128	27	61	Oluwafemi James Caleb et al. (2016)
86	27	59	Xiaoyin Yang et al. (2016)
136	24	116	Linda Höll et al. (2016)
247	24	54	Ali Jalali et al. (2017)
31	23	86	Bruna Leal Rodrigues et al. (2016)
380	23	50	Graziele G. Bovi et al. (2018)
314	22	112	Christian Ghidelli and Maria B. Pérez-Gago (2018)
505	22	63	Ce Wang et al. (2019)
280	21	133	Kenneth W. McMillin (2017)
397	21	90	Xiaoyin Yang et al. (2018)
411	20	64	Céline Matar et al. (2018)
283	19	47	Wenwen Wei et al. (2017)
47	19	30	Dimitrios Spanos et al. (2016)
41	18	49	Rolando Mendoza et al. (2016)

Source: Compiled by the authors.

## Data Availability

No-new data were created or analyzed in this study. Data sharing is not applicable to this article.
